# Effectiveness of BOX-PCR in Differentiating Genetic Relatedness among *Salmonella enterica* Serotype 4,[5],12:i:- Isolates from Hospitalized Patients and Minced Pork Samples in Northern Thailand

**DOI:** 10.1155/2019/5086240

**Published:** 2019-06-17

**Authors:** Kritchai Poonchareon, Chaiwat Pulsrikarn, Narong Nuanmuang, Phichaya Khamai

**Affiliations:** ^1^Division of Biochemistry, School of Medical Sciences, University of Phayao, 19 Moo 2, Tambon Maeka, Amphur Muang, Phayao 56000, Thailand; ^2^Department of Medical Sciences, WHO National Salmonella and Shigella Center, National Institute of Health, Ministry of Public Health, Tiwanond Road, Amphur Muang, Nonthaburi 11000, Thailand; ^3^Division of Clinical Microbiology, Department of Medical Technology, School of Allied Health Sciences, University of Phayao, 19 Moo 2, Tambon Maeka, Amphur Muang, Phayao 56000, Thailand

## Abstract

*Salmonella enterica* Serotype 4,[5],12:i:-, a monophasic variant of *S*. Typhimurium, with high virulence and multidrug resistance is distributed globally causing pathogenicity to both humans and domesticated animals. BOX-A1R-based repetitive extragenic palindromic-PCR (BOX)-PCR proved to be superior to three other repetitive element-based PCR typing methods, namely, enterobacterial repetitive intergenic consensus (ERIC)-, poly-trinucleotide (GTG)_5_-, and repetitive extragenic palindromic (REP)-PCR (carried out under a single optimized amplification condition), in differentiating genetic relatedness among *S*. 4,[5],12:i:- isolates from feces of hospitalized patients (*n*=12) and isolates from minced pork samples of *S*. 4,[5],12:i:- (*n*=6), *S*. Typhimurium (*n*=6), and *Salmonella* Serogroup B (*n*=4) collected from different regions of northern Thailand. Construction of phylogenetic trees from amplicon size patterns allowed allocation of *Salmonella* isolates into clusters of similar genetic relatedness, with BOX-PCR generating more unique clusters for each serotype than the other three typing methods. BOX-, (GTG)_5_-, and REP-PCR indicated significant genetic relatedness between *S*. 4,[5],12:i:- isolates 1 and 9 from hospitalized patients and *S*. 4,[5],12:i:- isolate en 29 from minced pork, suggesting a possible route of transmission. Thus, BOX-PCR provides a suitable molecular typing method for discriminating genetic relatedness among *Salmonella* spp. of the same and different serotypes and should be suitable for application in typing and tracking route of transmission in *Salmonella* outbreaks.

## 1. Introduction

Nontyphoidal *Salmonella* (NTS) is a cause of gastroenteritis, particularly in young children, the infection arising from consumption of contaminated food or unhygienic practices [1]. *Salmonella enterica* Serotype 4,[5],12:i:- is an emerging serotype with distribution worldwide and a significant infection rate of humans and domestic animals [2–5] including outbreaks in human populations of many countries [6].

Serologically related to *S.* Typhimurium, *S. enterica* 4,[5],12:i:- expresses O 4, 5, 12 antigens but not *flj*B (encoding phase 2 flagellum) due to defective phase switching [7]. The bacteria manifest multidrug resistance phenotype in many regions of the world including Thailand [8, 9]. The organism has been isolated from various animal species, e.g., chicken, cattle, swine, and turtles, and also from food items, such as raw poultry, pork, and pork sausage [10]. Furthermore, there exists evidence of genetic relatedness between *Salmonella* isolated from imported (Thai) pork products and (Danish) patients, suggesting an important route of *Salmonella* transmission across continents [11].

Molecular typing of *Salmonella* spp. is the usual assay performed to examine genetic relatedness, able to discriminate closely related *Salmonella* isolates, and reveal source-to-person strain transmission with sufficient precision to identify the specific source responsible for foodborne outbreaks [12]. A number of PCR-based typing techniques have been applied, such as direct sequencing of PCR amplicons, restriction fragment length polymorphism (RFLP)-PCR, amplified fragment length polymorphisms (AFLP)-PCR, random amplified polymorphic DNA (RAPD)-PCR, arbitrary primed (AP)-PCR, and pulsed-field gel-electrophoresis (PFGE)-PCR [13–16], the latter being the most popular technique and is commonly classified as the standard method due to its high discrimination and reproducibility, but the method requires specialized equipment, specific technical expertise, and lengthy (days) turn-around time. Other techniques have been developed to take advantage of known genetic elements, often noncoding intergenic repetitive sequences located in close proximity to one another, scattered across the genome, and using several PCR primers to amplify several families of repeated sequences. Examples of such methods include BOX-A1R-based **(**BOX)-, enterobacterial repetitive intergenic consensus (ERIC)-, poly-trinucleotide (GTG)_5_-, and repetitive extragenic palindromic (REP)-PCRs [16, 17].

The variability of genomic DNA sequences is identified by differences in sizes of the amplified fragments, and analysis of the different DNA fragment profiles can be performed using computer-assisted algorithms to cluster different patterns and construct phylogeny trees [18]. Those PCR primers can be utilized in different PCR protocols to evaluate their discrimination ability, sensitivity, and robustness [19].

The study sought to simplify identification of genetic relatedness with high discrimination between *S. enterica* 4,[5],12:i:- isolates from two different sources by comparing four different repetitive element-based PCR methods, namely, BOX-, ERIC-, (GTG)_5_-, and REP-PCR. Clustering power and discriminatory index of each technique were evaluated using the *S.* 4,[5],12:i:- isolates, together with *S*. Typhimurium and *S*. Serogroup B isolates. In addition, phylogenetic trees were constructed to determine relationship of clusters with other data sets, such as antibiogram profile and carriage of antibiotic-resistant genes.

## 2. Materials and Methods

### 2.1. Samples

**Figure 1:**
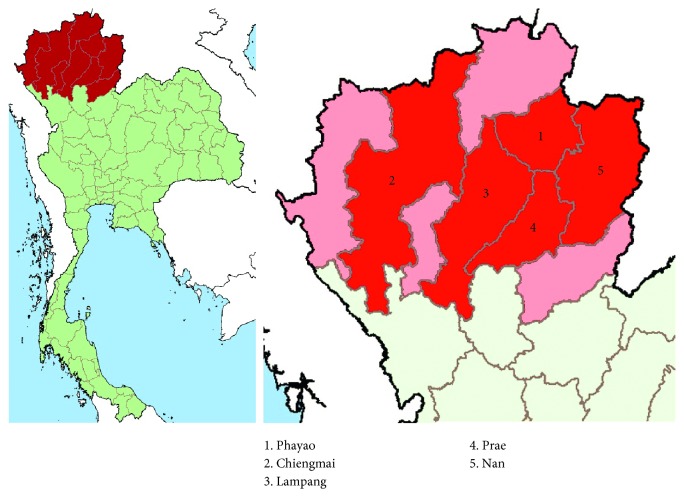
Map of Thailand showing northern region (red area, left panel) and provinces from which minced pork samples were obtained (right panel). Number of minced pork samples: Chiangmai, 3; Lampang, 3; Nan, 2; Phayao, 18; and Prae, 2.

*Salmonella* isolates consisted of *S*. 4,[5],12:i:- originally isolated from hospitalized patients (*n*=12) at Phayao Ram Hospital, Phayao province, during 2015–2017 [20], and *S*. 4,[5],12:i:- (*n*=6), *S*. Typhimurium (*n*=6), *S*. Serogroup B (*n*=3; *S*. Agona, *S.* Saintpaul, and *S.* Schwarzengrund), and one unknown *Salmonella* serotype from minced pork samples collected from retail markets in five different provinces of northern Thailand [21] (Figure 1), kept at 4°C until used.

### 2.2. Determination of Antibiotic Resistance Profile

Susceptibility to antibiotics of twelve *S*. 4,[5],12:i:- originally isolated from hospitalized patients was performed using a disk diffusion method following the Clinical and Laboratory Standards Institute (CLSI) [26] with ampicillin (AMP) l0 *μ*g, cefotaxime (CTX) 30 *μ*g, chloramphenicol (C), streptomycin (S) 10 *μ*g, sulphamethox/trimethoprim (SXT) 1.25 *μ*g/23.75 *μ*g, tetracycline (TE) 10 *μ*g, and colistin (COL) 10 *μ*g (Oxoid, Hampshire, UK). *Escherichia coli* ATCC 25922 was used as a negative control strain. The ESBL test was performed using the combination disk method according to CLSI criteria with both ceftazidime (30 *μ*g) and cefotaxime (30 *μ*g) alone and combined with clavulanic acid (10 *μ*g) (Oxoid, Hampshire, UK). In-house known ESBL-producing *Escherichia coli* and ESBL-negative *Escherichia coli* strains ATCC 25922 were used as controls.

### 2.3. BOX-, ERIC-, (GTG)_5_-, and REP-PCR Assays

DNA was extracted from *Salmonella* isolates as previously described [27]. In brief, the overnight culture (1 ml) was centrifuged, the pellet was washed twice with 400 *μ*l of TE buffer (10 mM Tris HCl, pH 8.0, 1 mM EDTA), and then the pellet was resuspended in 400 *μ*l of TE buffer. The resuspended solution was incubated at 80°C for 20 minutes. At room temperature, 50 *μ*L lysozyme (10 mg/mL) was added to the solution which was then incubated at 37°C for one hour with occasionally shaking followed by the addition of 75 *μ*L of 10% SDS/proteinase K solution with vigorous vertexing and incubation at 65°C for 10 minutes. Then, 100 *μ*L of 5 M NaCl and 100 *μ*L of prewarmed (65°C) CTAB/NaCl solution were added and additionally incubated at 65°C for 10 minutes. 750 *μ*l of chloroform/isoamyl alcohol (24 : 1) was added, and the solution was centrifuged for 5 minutes at 13,000 rpm at 4°C. The upper aqueous solution was collected, and then ethanol precipitation was performed. Finally, the pellet was resuspended with 50 *μ*l double-distilled water and the DNA solution was kept at −20°C until being further used.

To perform PCR reactions, each PCR mixture contained 0.1 *μ*L of DNA, different concentrations of each primer set (Table 1), 2 *μ*L of HOT FIREPol Blend Master Mix Plus 10 mM MgCl_2_ (Solis Biodye, Tartu, Estonia), and adjusted to 10 *μ*L with double-distilled water. Thermocycling was performed in Applied Biosystems (Thermo Fisher Scientific, Massachusetts, USA) as follows: 95°C for 15 minutes; 40 cycles of 95°C for 60 s; 54°C for 2 minutes; 72°C for 4 minutes; and a final step at 72°C for 10 minutes. Amplicons were separated by 4% agarose gel-electrophoresis, stained with RedSafe dye (INiRON, Washington, USA) and recorded using Molecular Imager Gel DOC™ XR+ (Bio-Rad, Berkeley, California, USA) equipped with Image Lab™ software as JPEG images at 300 dpi resolution.

### 2.4. Molecular Analysis of Major Beta-Lactamase Genes and *mcr-1*, 3, 4 Genes

Amplifications of different *bla* alleles and *mcr-1*, 3, 4 gene were performed by conventional monoplex or multiplex PCR using the primers (IDT, Singapore) listed in Table 1. The reaction mixture (10 *μ*l) contained 1 *μ*l of DNA, primer sets at concentration listed in Table 1, and 2 *μ*l of HOT FIREPol Blend Master Mix Plus 10 mM MgCl_2_ (Solis Biodye). In multiplex PCR 1 and 2, thermocycling was as follows: 95°C for 15 minutes; 40 cycles of 95°C for 40 s, 60°C for 40 s; 72°C for 1 minute; and a final step at 72°C for 7 minutes. Amplicons were visualized following 1.5% agarose gel electrophoresis by staining using RedSafe dye (INiRON, Washington, United States).

### 2.5. Amplicon Profile Analysis and Phylogenetic Tree Construction

Analysis of amplicon patterns generated by PCRs described above and construction of phylogenetic tree were carried out using curve-based algorithm (Pearson correlation) (Applied Maths, Sint-Martens-Latem, Belgium) to create a similarity scale and an unweighted pair group using arithmetic averages algorithm (UPGMA) for cluster analysis.

### 2.6. 3D Coordinate Space Window Construction

3D visualization of similarity to dataset of BOX-PCR clustering based on multidimensional scaling (MDS) was performed using a Metric algorithm (Applied Maths), and the coordinate space window was calculated based on the similarity matrix. Coordinate space window displayed each *S*. 4,[5],12:i:- isolates as dots in a cubic coordinate system and also as 3D spheres to enable visualization of 3D clustering in a realistic perspective.

### 2.7. Discriminatory Index Determination

In order to calculate the average probability that the molecular typing methods will assign a different type from two unrelated strains randomly sampled from the *Salmonella* isolates, a discriminatory index (*D*) was calculated at different levels of similarity index according to the formula [28]:

(1) $$\begin{array}{c} D=1-\frac{1}{N\left(N-1\right)}\overset{s}{\underset{j=1}{\sum }}x_{j}\left(x_{j}-1\right), \end{array}$$

where *D* = index of discriminatory power, *N* = number of unrelated strains tested, *S* = number of different types, and *x_j_* = number of strains belonging to *j* ^th^ type.

D value in a range of 0 (identical type) to 1.0 indicates that the typing method of interest is capable of distinguishing each member of a population from all other members of that population.

## 3. Results

### 3.1. Geographical Difference of Eighteen *S*. 4,[5],12:i:- Isolates Mostly Classified as Multidrug Resistant with Some Exhibiting Virulent ESBL Phenotype

*Salmonella* isolates in this study including *S*. Typhimurium (*n*=6) and *S.* 4,[5],12:i:- (*n*=18) as well as *Salmonella* Serogroup B (*n*=3) including *S*. Schwarzengrund, *S*. Agona, and *S*. Saintpaul and one unknown was either from the feces of hospitalized patients or minced pork collected from 5 different provinces of the northern Thailand (Figure 1). Most isolates of *S*. 4,[5],12:i:- showed multidrug resistance with five *Salmonella* isolates from hospitalized patients characterized as CTX-M group 1 producing *Salmonella* spp.; in addition, one *S*. Typhimurium isolate from minced pork in Nan province was characterized as CTX-M group 9 producing *Salmonella* spp. (Table 2). Three other *Salmonella* Serogroup B, *S*. Schwarzengrund, *S*. Agona, and *S*. Saintpaul, and one unknown, were included in the selection in attempt to generate out group cluster.

### 3.2. Molecular Typing of *S*. 4,[5],12:i:- Isolates from Hospitalized Patients and from Minced Pork Samples Collected in Northern Thailand

**Figure 2:**
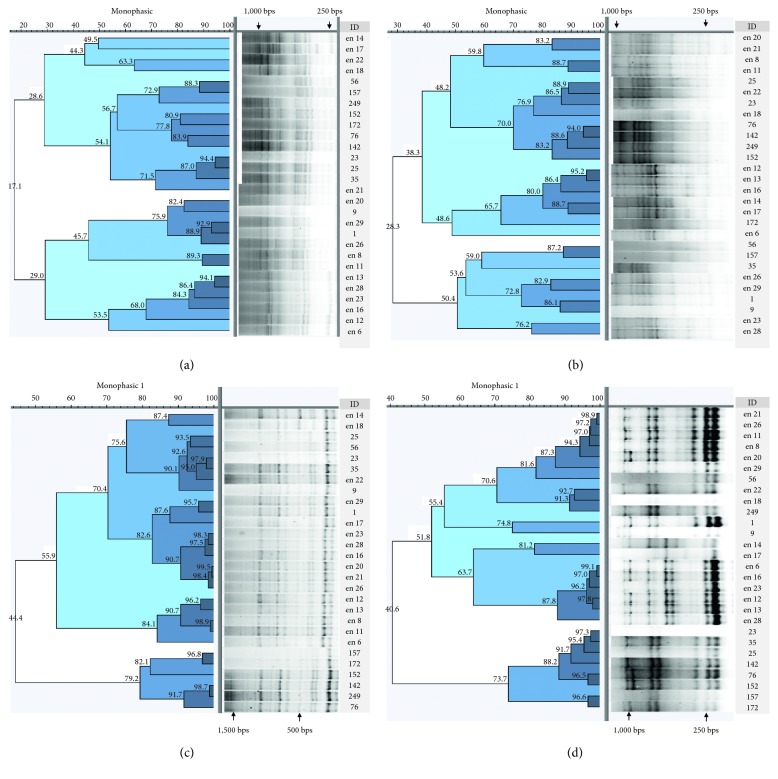
Amplicon profile and phylogenetic tree from BOX-PCR (a), GTG_5_-PCR (b), REP-PCR (c), and ERIC-PCR (d) of 28 *Salmonella* isolates collected in northern Thailand. PCRs were performed using the primer sets listed in Table 1. Phylogenetic trees were constructed using curve-based algorithm (Pearson correlation). The number at the branch node indicates percent amplicon profile similarity. Dark blue shade represents high cluster similarity. Light blue shade represents low cluster similarity. ID, *Salmonella* strains: en, from the minced pork sample; numeral, from feces of hospitalized patients.

Four different molecular typing methods, namely, BOX-, ERIC-, (GTG)_5_-, and REP-PCR, performed under the same optimized annealing temperature (54.0°C for 2 minutes), were applied to eighteen *S*. 4,[5],12:i:- isolates from hospitalized patients and from minced pork samples collected in northern Thailand, generating 9–28 amplicons of different sizes (100–1,500 bp) (Figure 2), with BOX-PCR demonstrating the highest mean number of amplicons, followed by REP-PCR, GTG_5_-PCR, and ERIC-PCR (Table 3). In order to compare the capability of each molecular typing method to differentiate among all *Salmonella* isolates, *D* was calculated from each constructed phylogenetic tree at three levels of similarity (50, 75, and 90%) using a curve-based algorithm (Pearson correlation) to create a similarity scale. A phylogenetic tree was constructed from each of the four PCR amplicon profiles (Figure 2), which showed BOX-PCR and GTG_5_-PCR with *D* > 0.9 at 75% and 90% similarity, while ERIC-PCR and REP-PCR have *D* > 0.9 only at 90% similarity (Table 4). Both the high average number of amplicons bands and high value of *D* suggest BOX-PCR and GTG_5_-PCR as better molecular typing methods than REP-PCR and ERIC-PCR in their capability to distinguish among closely genetically related *S*. 4,[5],12:i:- isolates from hospitalized patients and minced pork samples.

### 3.3. Ability of the Four Molecular Typing Methods to Differentiate Clusters of *Salmonella* Isolates with the Same Serotype

**Figure 3:**
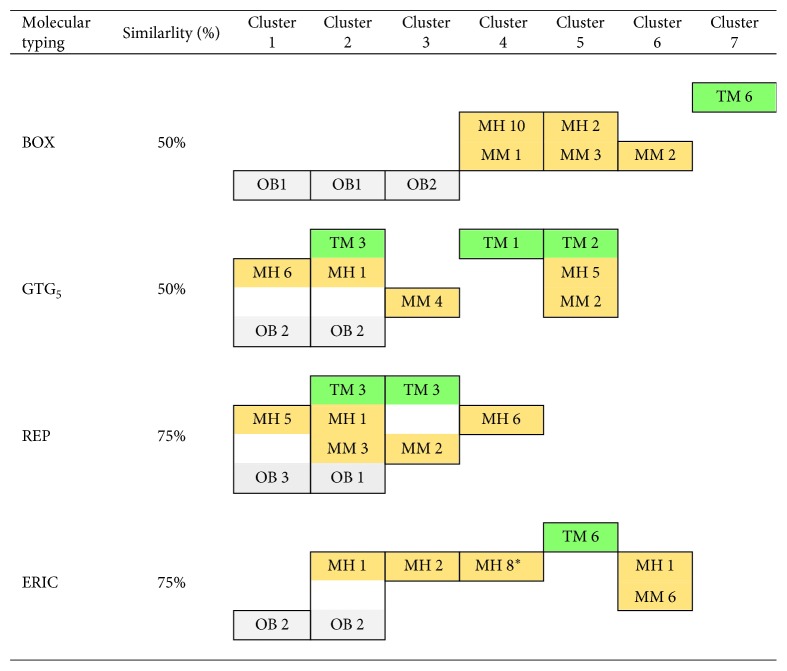
Clustering of *Salmonella* isolates collected from minced pork samples and feces of hospitalized patients in northern Thailand. Clustering of *Salmonella* isolates was performed using UPGMA algorithm at the indicated percent similarity of phylogeny obtained from Figure 2. The number of isolates in a cluster is indicated in color box. MH (yellow), *S*. 4,[5],12:i:- isolates from patients; MM (yellow), *S*. 4,[5],12:i:- isolates from minced pork; OB (gray), other *Salmonella* Serogroup B isolates from minced pork; TM (green), *S*. Typhimurium isolates from minced pork.

The UPGMA algorithm was applied to each molecular typing method in grouping into clusters of *Salmonella* spp. of the same serotype from same or different sources. At 50% similarity, BOX-PCR and GTG_5_-PCR were capable of differentiating *S*. Typhimurium and *S*. 4,[5],12:i:- isolates from minced pork into 2–4 clusters, while ERIC-PCR and REP-PCR placed *Salmonella* isolates of same serotype into one cluster each with *D* value = 0 (Table 4). Interestingly at 50% similarity, GTG_5_-PCR was capable of generating up to three clusters of six *S*. Typhimurium isolates with *D* value = 0.733 compared to one cluster for the other three PCR methods. At 80% similarity, all four molecular typing methods were able to differentiate the same serotype into different clusters except for ERIC-PCR that generated one cluster for six *S*. 4,[5],12:i:- isolates from minced pork. BOX-PCR and GTG_5_-PCR generated more clusters for each serotype from the same and different source(s) with *D* value = 0.6–0.7 (*S*. Typhimurium isolates) and 0.8–0.9 (*S*. 4,[5],12:i:- isolates from two sources) (Table 4). BOX-PCR generated the highest numbers of clusters of *S*. 4,[5],12:i:- isolates from hospitalized patients (*n*=12) with *D* value = 0.9091 and *S*. 4,[5],12:i:- isolates from minced pork (*n*=6) with *D* value = 0.8667. In addition, BOX-PCR effectively placed the four *Salmonella* Serogroup B isolates into their own cluster (Figure 3). BOX-PCR clearly was demonstrated to be the most suitable molecular typing method to group into clusters of similar genetic relatedness among *Salmonella* isolates of the same serotype both from the same source and from different sources.

### 3.4. Ability of BOX-, (GTG)_5_-, REP-, and ERIC-PCR to Differentiate Genetic Relatedness between *S.* 4,[5],12:i:- Isolates (en 26 and en 29) from Minced Pork Samples and Those (Isolates 1 and 9) from Hospitalized Patients

**Figure 4:**
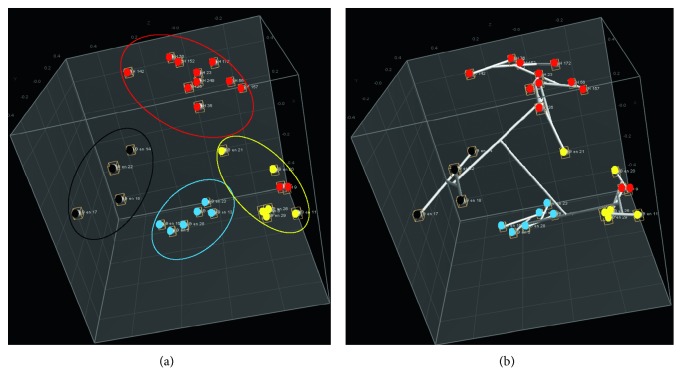
3D coordinate space window of genetic relatedness between two *Salmonella* 4,[5],12:i:- isolates from feces of hospitalized patients (ID 1 and 9) with groups of other *S*. 4,[5],12:i:- isolates from patients, *n*=12 (red dots), *S*. 4,[5],12:i:- isolates from minced pork, *n*=6, (yellow dots), *S*. Typhimurium isolates, *n*=6 (blue dots), and other *Salmonella* Serogroup B isolates, *n*=4 (black dots). Left panel: the 3D coordinate space window constructed using a multidimensional scaling (MDS) algorithm displays *Salmonella* isolates as clustered dots (color circle) in a cubic coordinate system. Right panel: the connected lines corresponding to distance of *Salmonella* isolates in clusters and between clusters were established in 3D coordinate space.

The genetic relatedness between *S*. 4,[5],12:i:- isolates from feces of hospitalized patients and minced pork samples was assessed by comparing the four constructed phylogeny trees (Figure 2). The analysis was performed by comparing the same cluster percent identity of each *S*. 4,[5],12:i:- isolate from minced pork samples to that of each *S*. 4,[5],12:i:- isolate from hospitalized patients. *S*. 4,[5],12:i:- isolates 1 and 9 from patients were genetically distant from *S*. 4,[5],12:i:- isolates from minced pork in all four PCR typing methods (Figure 2) explained with the results of cluster analysis (Table 5). BOX-, GTG_5_-, and REP-PCR indicated *S*. 4,[5],12:i:- isolates en 20, en 26, and en 29 from minced pork samples were of high genetic relatedness (>70%) to *S*. 4,[5],12:i:- isolates 1 and 9 from the patients, REP-PCR that the closest genetic relatedness (95.7%) was between *S*. 4,[5],12:i:- isolates 1 and en 29; and BOX-PCR that the genetic relatedness of *S*. 4,[5],12:i:- isolates 1 to also 9 to en 20 and en 29 was 82.4 and 92.9%, respectively. ERIC-PCR showed *S*. 4,[5],12:i:- isolates 56 had the closest genetic relatedness (81.6%) to all isolates from minced pork. The genetic relatedness between *S*. 4,[5],12:i:- isolates from two different sources could be clearly shown by the 3D coordinate space window, which demonstrated two *S*. 4,[5],12:i:- isolates of patients (ID 1 and 9) were in the cluster of *S*. 4,[5],12:i:- isolates from minced pork (Figure 4).

### 3.5. Relatedness of Phylogenetic Tree Constructed from BOX-PCR Amplicon Profiles with Antibiogram Profile and ESBL Production of *Salmonella* Isolates

**Figure 5:**
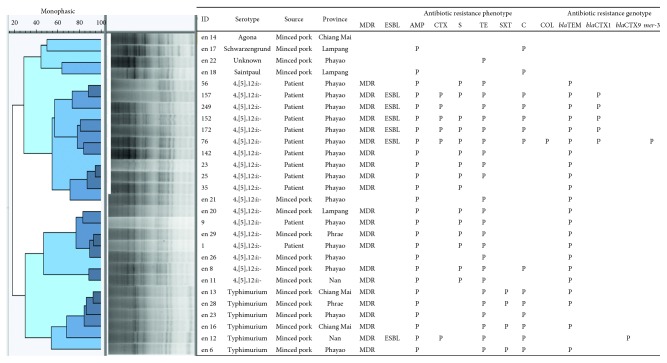
Association of *Salmonella* isolates with antibiogram profiles and ESBL production. Phylogenetic tree was constructed as described in the legend of Figure 2 using *S*. 4,[5],12:i:- isolates from feces of hospitalized patients (*n*=12) and from minced pork samples (*n*=6), *S*. Typhimurium isolates from minced pork (*n*=6), and other *Salmonella* Serogroup B isolates from minced pork (*n*=4). Antibiograms and ESBL-production properties of *Salmonella* isolates from minced pork were adapted from [21]. Dark blue shade represents high cluster similarity. Light blue shade represents low cluster similarity. AMP, ampicillin; C, chloramphenicol; COL, colistin; CTX, cefotaxime; S, streptomycin; SXT, sulfamethox/trimethoprim; TE, tetracyclin; ESBL, extended-spectrum beta lactamase; MDR, multidrug resistant; *p*, positive.

The phylogeny tree constructed from BOX-PCR amplicon profiles of *S*. 4,[5],12:i:- isolates from feces of hospitalized patients (*n*=12) and minced pork samples (*n*=6), *S*. Typhimurium isolates from minced pork samples (*n*=6), and other *Salmonella* Serogroup B isolates from minced pork samples (*n*=4) showed 50% similarity with three clusters of *S*. 4,[5],12:i:- isolates, one of *S*. Typhimurium isolates, and 3 of *S*. Serotype B isolates (Figure 5). The largest *S*. 4,[5],12:i:- cluster contained all isolates from hospitalized patients, and the other two clusters included mainly minced pork isolates in one and the four *S*. Serogroup B isolates in the other. According to the previous antibiogram profiles of *Salmonella* spp. from minced pork [21] and from hospitalized patients in this study, the majority of *S*. 4,[5],12:i:- and *S*. Typhimurium isolates were multidrug-resistant with specific antibiogram profile corresponding to the serotype, e.g., *S*. Typhimurium was mainly resistant to ampicillin, chloramphenicol, and tetracycline (AMP/C/TE) with optional sulphamethox/trimethoprim (SXT), while *S*. 4,[5],12:i:- mainly to ampicillin, streptomycin, and tetracycline (AMP/TE/S). All ESBL-producing *S*. 4,[5],12:i:- isolates from patients were clustered together and apart from ESBL-producing isolates of minced pork, but all with the same antibiogram (AMP/TE/S/C/CTX). There was complete linkage between chloramphenicol resistance and ESBL-producing *S*. 4,[5],12:i:- isolates, *bla*CTX-M group 1 being the predominant determinant. From cluster analysis (Figure 2), *S*. 4,[5],12:i:- isolates in the cluster consisting of isolates 1 and 9 from patients and from minced pork showed the common shared antibiogram (AMP/TE/S). One ESBL-producing *S*. 4,[5],12:i:- resistant to meristin through acquisition of *mcr*-3 gene was also observed (Figure 5).

## 4. Discussion

Many types of short-interspersed repetitive DNA sequences have been identified in prokaryotic genomes [24], and BOX elements are characterized as being conserved among diverse bacterial species and serve as potential targets for identifying genetic relatedness in both Gram-negative and Gram-positive bacteria, such as *Aeromonas* spp. [29], *Escherichia coli* [30, 31], and *Streptococcus pneumoniae* [32].

The constructed phylogeny tree from BOX-PCR typing effectively differentiated genetic relatedness of *S*. 4,[5],12:i:- isolates as well as grouping them into different clusters according to their origin, feces of hospitalized patient, or minced pork sample. Previous studies in Germany employing PFGE technique and phage typing were successfully performed to monitor the genetic relatedness among *S*. 4,[5],12:i:- isolated from pig, pork meat, and humans [33]. BOX-, GTG_5_-, and REP-PCR similarly identified two isolates from hospitalized patients (ID 1 and 9) with high genetic relatedness to isolates from minced pork, suggesting the possibility that (some) *Salmonella* isolates causing human infection could have come from contaminated food (minced pork) as traditional food of northern Thai food often contains raw meat, such as raw spicy minced pork. Many studies have shown contaminated raw meat and poultry are causes of *Salmonella* transmission if there is a lapse in food safety practices, leading to increased risks in salmonellosis outbreaks [34].

Repetitive element-based (RE)-PCR assays were shown to be capable of typing 80 serotypes and five isolates previously not typeable as well as generating amplicon profile heterogeneity within some serotypes [35]. RE-PCR was shown to be a better serotyping method over traditional serotyping of *Salmonella* isolates during outbreak investigations [36]. Furthermore, the greater discriminative ability of RE-PCR over the standard PFGE protocol indicates the former to be the preferred method to detect *Salmonella* transmission links [37]. In addition, composite of a number of RE-PCR methods offer even more discriminatory power in estimation of genetic relatedness stemming from different independent genetic information obtained from the different RE-PCR primers [37]. RE-PCR performs better than MLST in subtyping *Salmonella* Enteritidis isolates of food and human origin [38].

Virulent ESBL-producing *S*. 4,[5],12:i:- isolates from feces of hospitalize patients highly shared genetic relatedness and formed a unique cluster, with their antibiograms indicating acquisition of *bla*CTX group 1 as reported in many countries [39, 40]. To the best of our best knowledge, ESBL-producing *S*. 4,[5],12:i:- isolates resistant to meristin and harboring *mcr*-3 gene is the first observed in northern Thailand, which poses the risk of traveler's diarrhea to those returning after travelling in this region of the country [41]. In addition, to the best of our knowledge, this is the first study in which four different RE-PCR typing methods were compared in evaluating genetic relatedness among *S*. 4,[5],12:i:- isolates from different sources and geography.

## 5. Conclusion

The simple BOX-PCR typing method is effective in differentiating genetic relatedness of *S*. 4,[5],12:i:- isolates from feces of hospitalized patients in Phayao province, northern Thailand, and those from minced pork samples obtained at different locations in the same region of the country and should be adopted in tracking transmission during *Salmonella* outbreaks.

## Figures and Tables

**Table 1:** Primers used in this study.

Primer	Genes	Sequence (5′ ⟶ 3′)	Size of PCR product (bps)	Primer concentration (pmol/*μ*l)	Reference
Antibiotic-resistant determinants					
*Multiplex 1 blaTEM variants including blaTEM-1 and blaTEM-2, blaSHV variants including blaSHV-1, and blaOXA-1-like including blaOXA-1, blaOXA-4, and blaOXA-30*					
blaTEM_f	*bla*TEM	CATTTCCGTGTCGCCCTTATTC	800	0.4	[22]
blaTEM_r		CGTTCATCCATAGTTGCCTGAC		0.4	[22]
blaSHV_f	*bla*SHV	AGCCGCTTGAGCAAATTAAAC	713	0.4	[22]
blaSHV_r		ATCCCGCAGATAAATCACCAC		0.4	[22]
blaOXA_f	*bla*OXA	GGCACCAGATTCAACTTTCAAG	564	0.4	[22]
blaOXA_r		GACCCCAAGTTTCCTGTAAGTG		0.4	[22]

*Multiplex 2 blaCTX-M group 1 and group 9: variants of blaCTX-M group 1 including blaCTX-M-1, blaCTX-M-3, and blaCTX-M-15 and variants of blaCTX-M group 9 including blaCTX-M-9 and blaCTX-M-14*					
CTX 1_f	*bla*CTX-M group 1	TTAGGAARTGTGCCGCTGYA^b^	688	0.4	[22]
CTX 1_r		CGATATCGTTGGTGGTRCCAT^b^		0.2	[22]
CTX 9_f	*bla*CTX-M group 9	TCAAGCCTGCCGATCTGGT	561	0.4	[22]
CTX 9_r		TGATTCTCGCCGCTGAAG		0.4	[22]

*Multiplex 3 blaCTX-M group 1 and group 9*					
CTX 1_f	*bla*CTX-M group 1	TTAGGAARTGTGCCGCTGYA^b^	688	0.4	[22]
CTX 1_r		CGATATCGTTGGTGGTRCCAT^b^		0.2	[22]
CTX 9_f	*bla*CTX-M group 9	TCAAGCCTGCCGATCTGGT	561	0.4	[22]
CTX 9_r		TGATTCTCGCCGCTGAAG		0.4	[22]

*Multiplex 4 mcr variants including mcr 1, 3, and 4*					
mcr 1_f	*mcr-1*	AGTCCGTTTGTTCTTGTGGC	320	0.25	[23]
mcr 1_r		AGATCCTTGGTCTCGGCTTG		0.25	[23]
mcr 3_f	*mcr-3*	AAATAAAAATTGTTCCGCTTATG	929	0.25	[23]
mcr 3_r		AATGGAGATCCCCGTTTTT		0.25	[23]
mcr 4_f	*mcr-4*	TCACTTTCATCACTGCGTTG	1116	0.25	[23]
mcr 4_r		TTGGTCCATGACTACCAATG		0.25	[23]

*Molecular typing*					
ERIC_f	ERIC-PCR	ATGTAAGCTCCTGGGGATTCAC		25	[24]
ERIC_r		AAGTAAGTGACTGGGGTGAGCG		25	[24]
GTG_fr	GTG_5_-PCR	GTGGTGGTGGTGGTG		25	[15]
BOXA1R_fr	BOX-PCR	CTACGGCAAGGCGACGCTGACG		20	[25]
REP_f	REP-PCR	IIIGCGCCGICATCAGGC		25	[16]
REP_r		ACGTCTTATCAGGCCTAC		25	[16]

^b^Y = T or C; R = A or G; S = G or C; D = A or G or T.

**Table 2:** The descriptive data of 28 *Salmonella* spp. isolates indicating their serotypes, locations of collection, multidrug resistance, and extended-spectrum beta lactamase (ESBL).

Number	ID^a^	Serotype	Source	Locality (province)^b^	Antibiotic resistance	ESBL
1	en 6	Typhimurium	Minced pork	Phayao	AMP/TE/SXT/C^*∗*^	
2	en 12	Typhimurium	Minced pork	Nan	AMP/CTX/TE/C^*∗*^	P
3	en 13	Typhimurium	Minced pork	Chiang Mai	AMP/TE/SXT/C^*∗*^	
4	en 16	Typhimurium	Minced pork	Chiang Mai	AMP/TE/SXT/C^*∗*^	
5	en 23	Typhimurium	Minced pork	Phayao	AMP/TE/C^*∗*^	
6	en 28	Typhimurium	Minced pork	Phrae	AMP/TE/SXT/C^*∗*^	
7	en 8	4,[5],12:i:-	Minced pork	Phayao	AMP/S/TE/C^*∗*^	
8	en 11	4,[5],12:i:-	Minced pork	Nan	AMP/S/TE^*∗*^	
9	en 20	4,[5],12:i:-	Minced pork	Lampang	AMP/S/TE^*∗*^	
10	en 21	4,[5],12:i:-	Minced pork	Phayao	AMP/TE	
11	en 26	4,[5],12:i:-	Minced pork	Phayao	AMP/TE	
12	en 29	4,[5],12:i:-	Minced pork	Phrae	AMP/S/TE^*∗*^	
13	1	4,[5],12:i:-	Hospitalized patient	Phayao	AMP/S/TE^*∗*^	
14	9	4,[5],12:i:-	Hospitalized patient	Phayao	AMP/S/TE^*∗*^	
15	23	4,[5],12:i:-	Hospitalized patient	Phayao	AMP/S/TE^*∗*^	
16	25	4,[5],12:i:-	Hospitalized patient	Phayao	AMP/S/TE^*∗*^	
17	35	4,[5],12:i:-	Hospitalized patient	Phayao	AMP/S	
18	56	4,[5],12:i:-	Hospitalized patient	Phayao	AMP/S/TE^*∗*^	
19	76	4,[5],12:i:-	Hospitalized patient	Phayao	AMP/CTX/S/TE/C/COL^*∗*^	P
20	142	4,[5],12:i:-	Hospitalized patient	Phayao	AMP/S/TE^*∗*^	
21	152	4,[5],12:i:-	Hospitalized patient	Phayao	AMP/CTX/S/TE/C^*∗*^	P
22	157	4,[5],12:i:-	Hospitalized patient	Phayao	AMP/CTX/S/TE/C^*∗*^	P
23	172	4,[5],12:i:-	Hospitalized patient	Phayao	AMP/CTX/S/TE/C^*∗*^	P
24	249	4,[5],12:i:-	Hospitalized patient	Phayao	AMP/CTX/TE/C^*∗*^	P
25	en 22	Unknown	Minced pork	Phayao	TE	
26	en 14	Agona	Minced pork	Chiang Mai	AMP	
27	en 17	Schwarzengrund	Minced pork	Lampang	AMP/C	
28	en 18	Saintpaul	Minced pork	Lampang	AMP/C	

ID^a^, *Salmonella* strains: en, from the minced pork sample; numeral, from feces of hospitalized patients. ^b^ Figure 1. MDR: multidrug resistant; AMP: ampicillin; CTX: cefotaxime; TE: tetracyclin; S: streptomycin; SXT: sulphamethox/trimethoprim; C: chloramphenicol; COL: colistin. “P” *Salmonella* isolates showed positive characteristics. Note that only the antibiotic profile of *Salmonella* isolates from the hospitalized patient was conducted in this experiment.

**Table 3:** Amplicons generated by the four molecular typing methods and discriminatory index.

Molecular typing method	Number of bands (min–av–max)	Size (bp)	Discriminatory index^*∗*^		
			50%	75%	90%
BOX-PCR	19–24.07–28	200–1000	0.7804	0.9286	0.9921
GTG_5_-PCR	9–18.01–23	100–1500	0.7751	0.9259	0.9947
REP-PCR	14–18.75–21	250–1000	0.3492	0.7646	0.8915
ERIC-PCR	11–13.78–16	100–1200	0.4233	0.7963	0.9180

^*∗*^From Figure 2 at various percent similarity of amplicon profile. av: average; max: maximum; min: minimum.

**Table 4:** Differentiation into clusters by the four molecular typing methods of *Salmonella* isolates of the same serotype collected from the same source and two different sources.

Percent similarity^a^	Serotype (number of isolates)	Source	Number of clusters^a^ (number of isolates in each cluster), discriminatory index^b^			
			BOX-PCR	GTG_5_-PCR	REP-PCR	ERIC-PCR
50%	Typhimurium (6)	Minced pork	1 (6), 0	3 (3, 2, 1), 0.733	1 (6), 0	1 (6), 0
	*S*. 4,[5],12:i:- (6)	Minced pork	3 (3, 2, 1), 0.7333	2 (4, 2), 0.5333	1 (6), 0	1 (6), 0
	*S*. 4,[5],12:i:- (12)	Hospitalized patients	2 (10, 2), 0.303	3 (6, 5, 1), 0.6212	2 (6, 6), 0.5455	2 (8, 4), 0.4848

80%	Typhimurium (6)	Minced pork	3 (4, 1, 1), 0.6	3 (3, 2, 1), 0.7333	2 (3, 3), 0.6	1 (6), 0
	*S*. 4,[5],12:i:- (6)	Minced pork	4 (2, 2, 1, 1), 0.8667	3 (2, 2, 2), 0.8	2 (4, 2), 0.5333	1 (6), 0
	*S*. 4,[5],12:i:- (12)	Hospitalized patients	7 (3, 2, 2, 2, 1, 1, 1), 0.9091	6 (4, 2, 2, 2, 1, 1), 0.8636	4 (5, 3, 3, 1), 0.7576	6 (6, 2, 1, 1, 1, 1), 0.7576

^a^From phylogenetic tree (Figure 2). ^b^[28].

**Table 5:** Genetic relatedness of *S*. 4,[5],12:i:- isolates from minced pork and those from feces of hospitalized patients.

Strain ID^a^	Locality (province)^b^	Strain ID from patient^c^ (percent genetic similarity)^d^			
		BOX-PCR	GTG_5_-PCR	REP-PCR	ERIC-PCR
en 8	Phayao	1, 9 (45.7%)	23, 25, 76, 142, 152, 249 (48.2%)	1, 9, 23, 25, 35, 56 (55.9%)	56 (81.6%)
en 21	Phayao	35, 25, 23 (71.5%)	23, 25, 76, 142, 152, 249 (48.2%)	1 (87.6%)	56 (81.6%)
en 26	Phayao	1 (88.9%)	1, 9 (72.8%)	1 (87.6%)	56 (81.6%)
en 11	Nan	1, 9 (45.7%)	23, 25, 76, 142, 152, 249 (48.2%)	1, 9, 23, 25, 35, 56 (55.9%)	56 (81.6%)
en 20	Lampang	9 (82.4%)	23, 25, 76, 142, 152, 249 (48.2%)	1 (87.6%)	56 (81.6%)
en 29	Phrae	1 (92.9%)	1, 9 (72.8%)	1 (95.7%)	56 (81.6%)

^a^From minced pork. ^b^ Figure 1. ^c^In the same cluster as minced pork sample (Figure 2). ^d^Highest value observed from the maximal similarity that each strain ID from minced pork shared with strain ID from patients in Figure 2.

## Data Availability

The original gel pictures used to support the findings of this study are included within the supplementary information file.

## Supplementary Materials

Supplementary Materials These supplementary figures provided the original gel pictures of 28 *Salmonella* spp. performing each Rep PCR in this study.
