# Supplementary material for: Effectiveness of BOX-PCR in Differentiating Genetic Relatedness among Salmonella enterica Serotype 4,[5],12:i:- Isolates from Hospitalized Patients and Minced Pork Samples in Northern Thailand
Source: Int J Microbiol. 2019 Jun 17;2019:5086240. doi: 10.1155/2019/5086240 (PMC6604291; doi:10.1155/2019/5086240)
Supplement: Supplementary Materials — These supplementary figures provided the original gel pictures of 28 Salmonella spp. performing each Rep PCR in this study. [file 5086240.f1.pptx]

## Slide 1
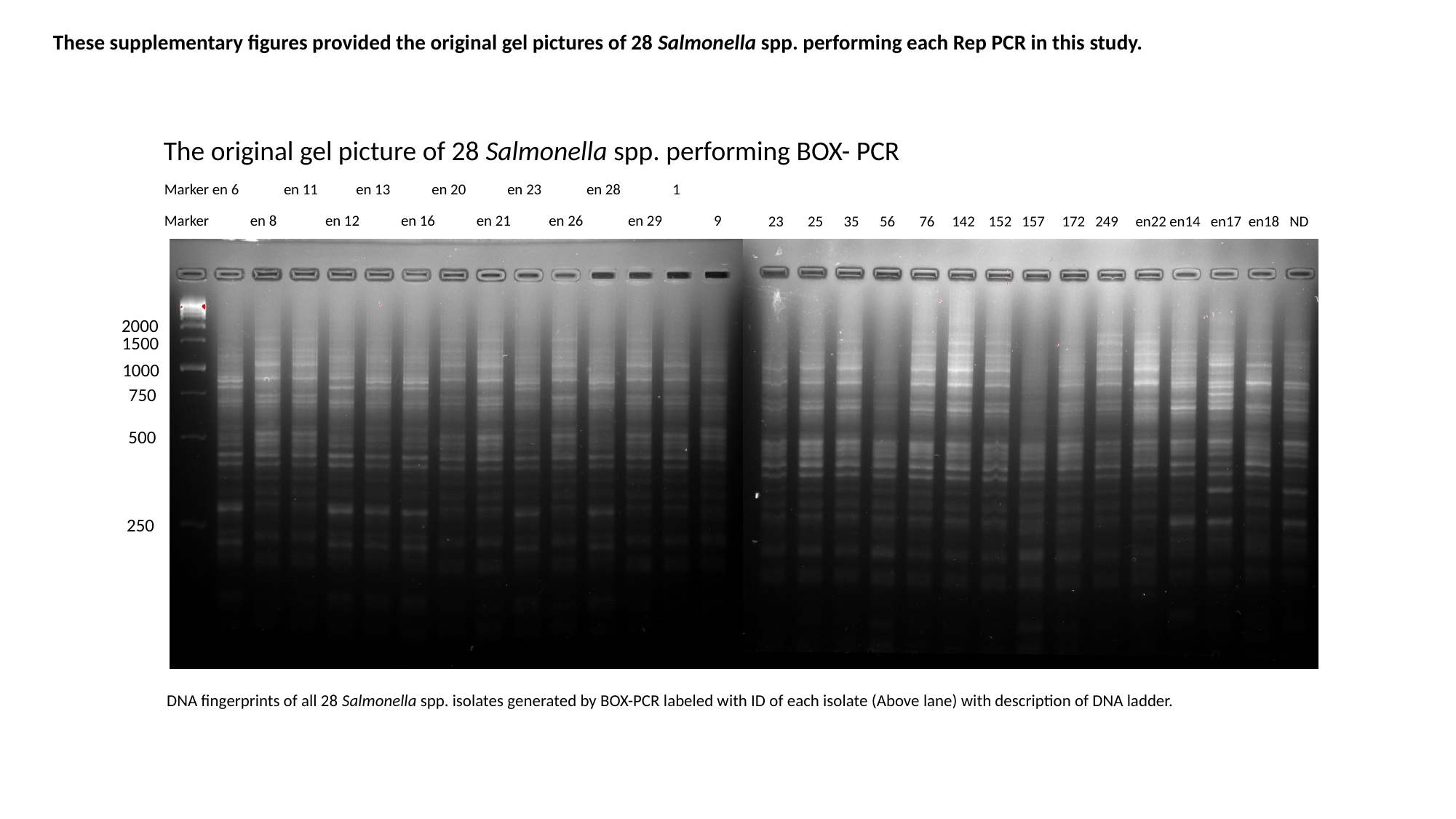

These supplementary figures provided the original gel pictures of 28 Salmonella spp. performing each Rep PCR in this study.
The original gel picture of 28 Salmonella spp. performing BOX- PCR
Marker en 6 en 11 en 13 en 20 en 23 en 28 1
Marker en 8 en 12 en 16 en 21 en 26 en 29 9
23 25 35 56 76 142 152 157 172 249 en22 en14 en17 en18 ND
2000
1500
1000
750
500
250
DNA fingerprints of all 28 Salmonella spp. isolates generated by BOX-PCR labeled with ID of each isolate (Above lane) with description of DNA ladder.

## Slide 2
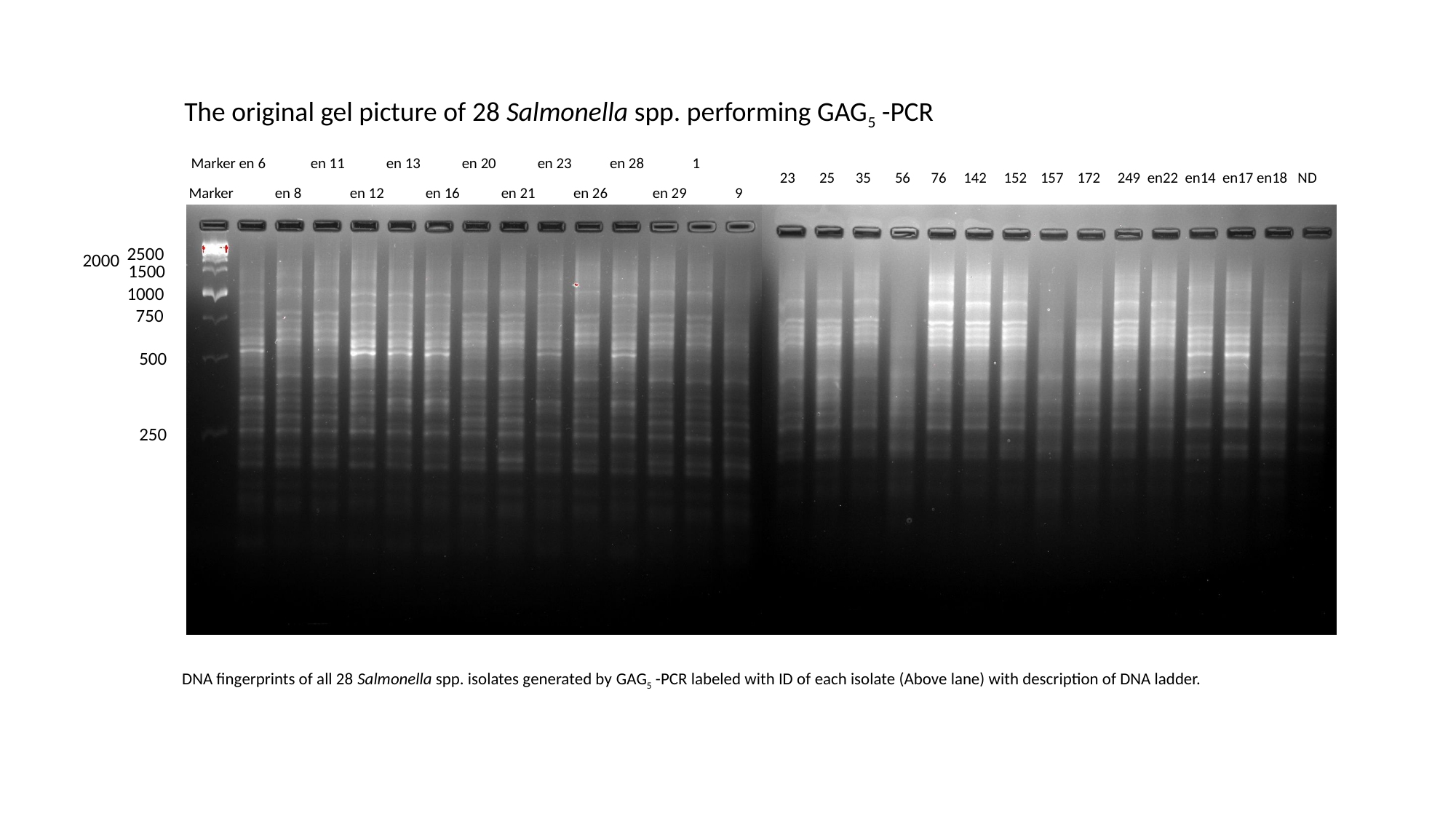

The original gel picture of 28 Salmonella spp. performing GAG5 -PCR
Marker en 6 en 11 en 13 en 20 en 23 en 28 1
23 25 35 56 76 142 152 157 172 249 en22 en14 en17 en18 ND
Marker en 8 en 12 en 16 en 21 en 26 en 29 9
2500
2000
1500
1000
750
500
250
DNA fingerprints of all 28 Salmonella spp. isolates generated by GAG5 -PCR labeled with ID of each isolate (Above lane) with description of DNA ladder.

## Slide 3
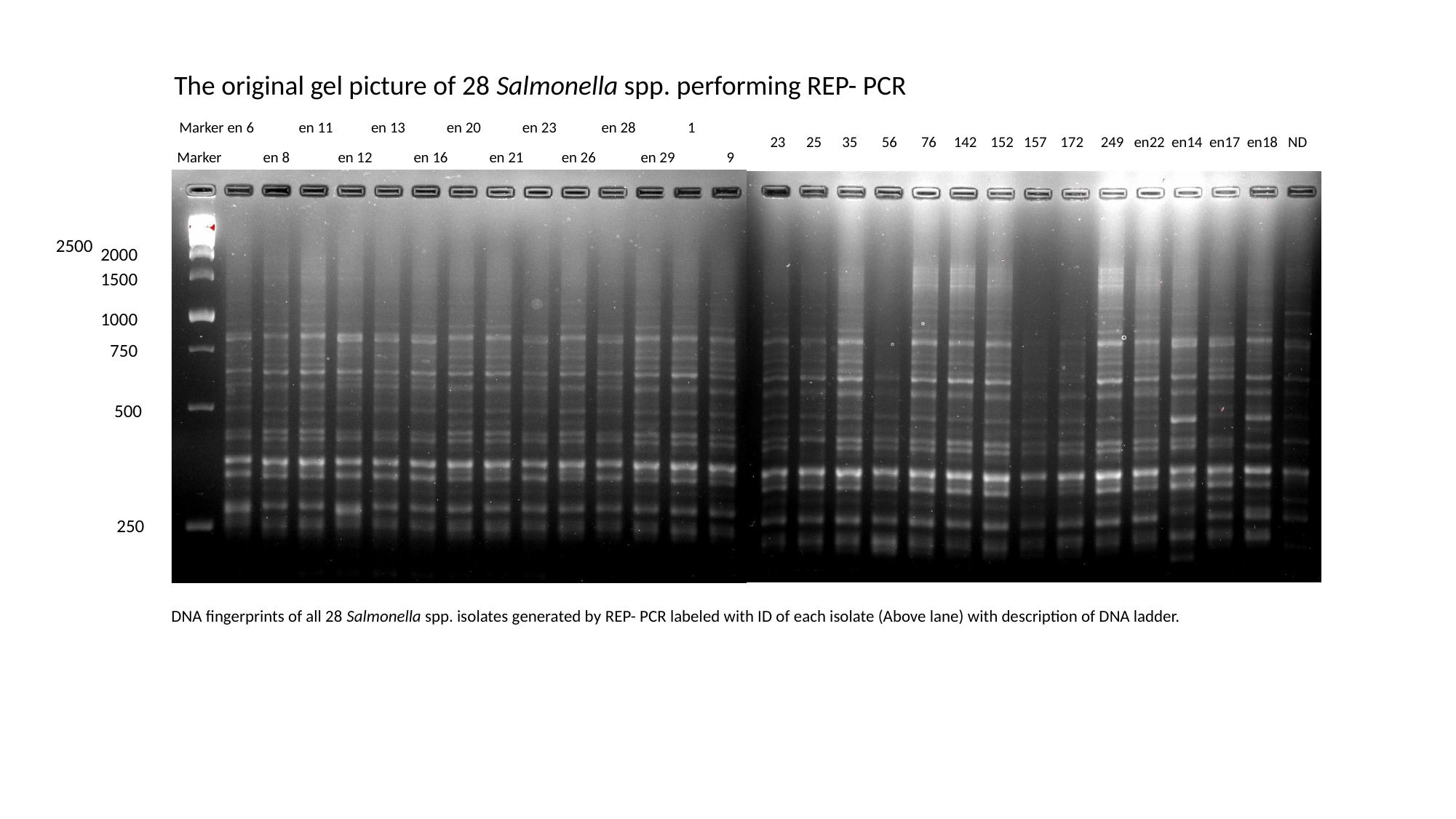

The original gel picture of 28 Salmonella spp. performing REP- PCR
Marker en 6 en 11 en 13 en 20 en 23 en 28 1
23 25 35 56 76 142 152 157 172 249 en22 en14 en17 en18 ND
Marker en 8 en 12 en 16 en 21 en 26 en 29 9
2500
2000
1500
1000
750
500
250
DNA fingerprints of all 28 Salmonella spp. isolates generated by REP- PCR labeled with ID of each isolate (Above lane) with description of DNA ladder.

## Slide 4
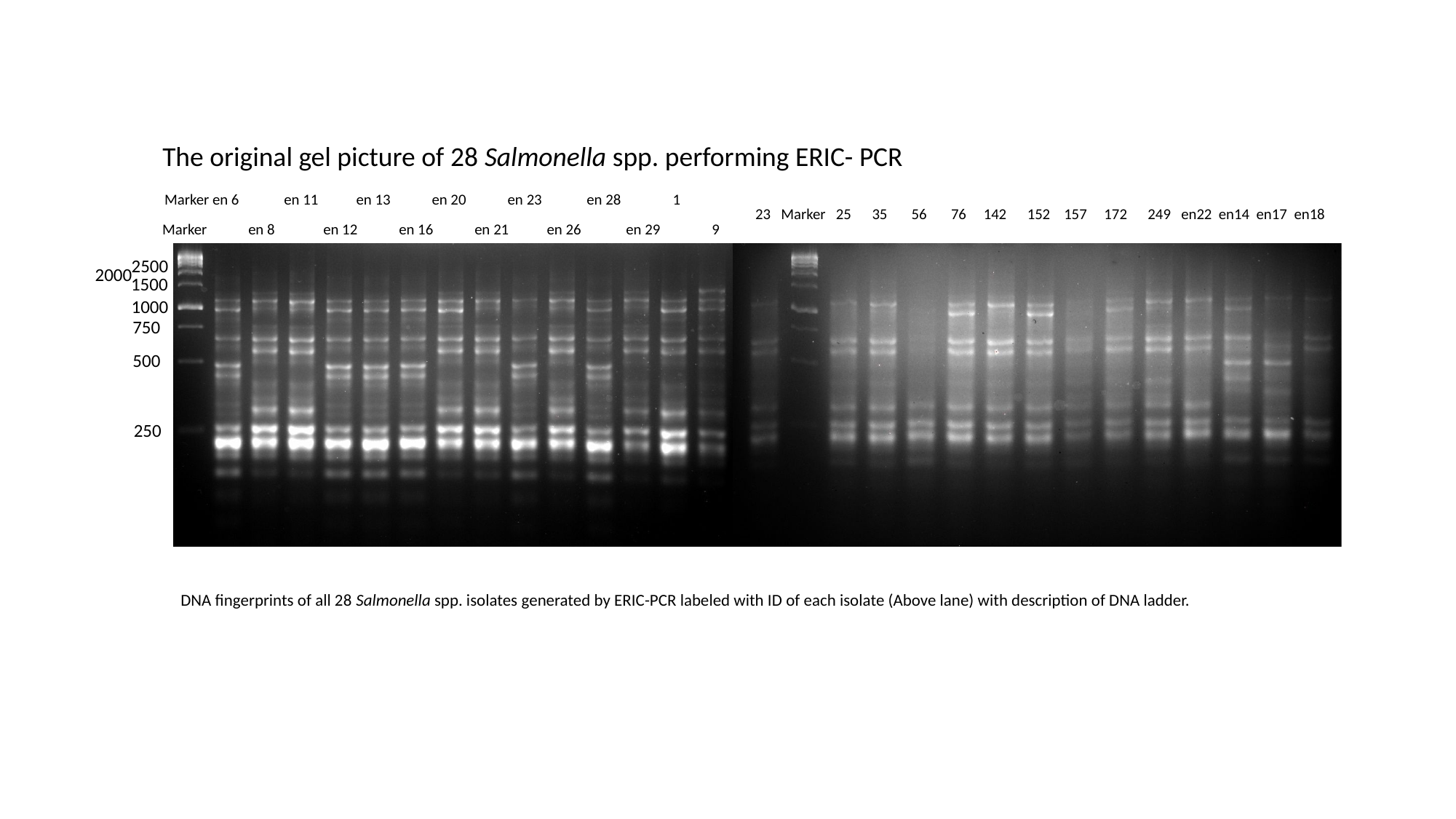

The original gel picture of 28 Salmonella spp. performing ERIC- PCR
Marker en 6 en 11 en 13 en 20 en 23 en 28 1
23 Marker 25 35 56 76 142 152 157 172 249 en22 en14 en17 en18
Marker en 8 en 12 en 16 en 21 en 26 en 29 9
2500
2000
1500
1000
750
500
250
DNA fingerprints of all 28 Salmonella spp. isolates generated by ERIC-PCR labeled with ID of each isolate (Above lane) with description of DNA ladder.
